# Transcriptome Analysis of lncRNA–mRNA Interactions in Chronic Atrophic Gastritis

**DOI:** 10.3389/fgene.2020.612951

**Published:** 2021-01-11

**Authors:** Yang Chao, Jingpeng Jin, Liqiang Wang, Xiya Jin, Lei Yang, Bin Zhang

**Affiliations:** Department of Gastroendoscopy, China-Japan Union Hospital of Jilin University, Changchun, China

**Keywords:** chronic atrophic gastritis, chronic non-atrophic gastritis, lncRNA, mRNA, biomarker

## Abstract

The aim of this study was to identify prognosis-related differentially expressed lncRNAs and mRNAs in chronic atrophic gastritis (CAG). By analysis of high-throughput whole-transcriptome sequencing data, the levels of lncRNAs and mRNAs between CAG and chronic non-atrophic gastritis were compared pairwisely. In total, 97,282 lncRNA transcripts and 20,307 mRNA transcripts were acquired, including 50 upregulated and 66 downregulated lncRNAs and 377 upregulated and 763 downregulated mRNAs in CAG (*p* < 0.05, fold change ≥ 2). Moreover, the interactions of the differentially expressed genes in CAG were investigated by gene ontology enrichment analysis, showing that the enriched genes are involved in many biological processes, such as MAP kinase activity, heat generation, and protein modification processes. Through the construction of co-expression networks of the differentially expressed genes in CAG, three critical lncRNAs nodes were identified as potential key factors in CAG. Eight mRNAs common in both the co-expression network and the protein–protein interaction network were selected via Venn analysis, including DGKA, EIF6, HKDC1, DHRS11, 1, KRT15, TESPA1, and CDHR2. Finally, the expression levels of five differentially expressed lncRNAs in CAG were confirmed by quantitative real-time polymerase chain reaction. In conclusion, this study presents novel promising biomarkers for the diagnosis of CAG.

## Introduction

Chronic atrophic gastritis (CAG) involves chronic inflammation of the gastric mucosa that is characterized by atrophy of the gastric mucosa, loss of intrinsic glands, and intestinal metaphasia. The most common symptoms of CAG include upper abdominal pain, abdominal distension, abdominal discomfort, and anorexia. Chronic gastritis is divided into two categories, i.e., non-atrophic and atrophic. The process of chronic non-atrophic gastritis (CNAG) has been proposed to occur through three stages: glandular atrophy, metaphasia dysplasia, and, ultimately, gastric cancer (GC; Correa, 1988, 1992). Although the incidence and mortality of GC in China are falling, GC is still the second leading cause of death in China, according to reports from the National Central Cancer Registry of China (Chen et al., 2016; Bray et al., 2018). As a precancerous lesion of GC, CAG has recently been given increasing attention because of the essential role of the early diagnosis and treatment of CAG in the prevention of gastric carcinogenesis. However, due to unobvious clinical manifestations and reduced tolerance to invasive examinations in CAG patients, the early diagnosis of CAG remains difficult in clinical practice.

Currently, the diagnosis of CAG is commonly made by endoscopic examination and pathological features. However, the assessment of mucosal atrophy by endoscopy and histology has a few limitations, such as the weak correlation between endoscopic and histopathological findings, disagreements between endoscopic diagnoses made by different observers, and inaccurate orientation and misdiagnosis caused by histopathological examination of inappropriate sections (Dixon et al., 1996). To ascertain the existence and location of atrophy, the use of pepsinogen (PG) I or II in the examination may be helpful; however, this method has not been widely accepted because of the low stability of PG (Syrjanen, 2016; Fang et al., 2018). Therefore, the discovery of new diagnostic biomarkers of CAG with high sensitivity and specificity for the early diagnosis of CAG is urgently needed.

Non-coding RNAs (ncRNAs) are a type of functional RNA molecules that are not translated into protein. Based on their size, ncRNAs are divided into small ncRNA (sncRNA) of approximately 22 nucleotides in length and long ncRNA (lncRNA) with lengths longer than 200 nucleotides (Frith et al., 2006; Dinger et al., 2008; Perkel, 2013). LncRNAs are regarded as critical regulators of gene expression at both the transcriptional and post-transcriptional levels (Wilusz et al., 2008; Mercer et al., 2009; Ponting et al., 2009; Chen and Carmichael, 2010; Hung and Chang, 2010; Flynn and Chang, 2014). It has been reported that lncRNA can be used as a *cis-*acting factor to regulate the expression of nearby genes in the genome; for example, lncRNA Xist can be used as a *cis-*acting element to silence the X chromosome (Hall and Lawrence, 2010; Pollex and Heard, 2012). LncRNA also can act as a *trans*-acting element to regulate gene transcription. Rinn et al. (2007) found that long-stranded non-coding RNA-HOTAIR can directly bind to PcG protein, thereby recruiting PRC2 complex to the location of HOXD gene, thus realizing the regulation of HOXD gene. LncRNA can often bind protein molecules through specific elements and affect the molecular activity and intracellular localization of proteins. For example, the highly conserved long-stranded non-coding RNAEvf-2 can form a RNA–protein complex with the transcription factor Dlx2. Using luciferase reporter plasmid, a study found that the transcriptional activity of Dlx2 depended on the transcription of Evf-2 (Feng et al., 2006). Furthermore, lncRNA may affect the post-transcriptional modification of messenger RNA. Gong et al. found that some long-stranded non-coding RNA containing Alu are the key regulatory molecules that mediate the binding and interaction of these messenger RNA by STAU-1 (Gong and Maquat, 2011). On the other hand, lncRNAs have been reported as molecular biomarkers for the diagnosis of many diseases (Tinzl et al., 2004; Wang et al., 2006, 2014; Xie et al., 2013; Pang et al., 2014; Shao et al., 2014; Svoboda et al., 2014; Zhao et al., 2014, 2020; Li et al., 2015, 2019; Tang et al., 2015; Zhou et al., 2015, 2019; Cao et al., 2019). Since lncRNAs have higher tissue specificity than protein-coding mRNAs (Cabili et al., 2011; Derrien et al., 2012; Molyneaux et al., 2015), it is feasible to develop novel diagnostic biomarkers from lncRNA. Thus, lncRNAs might be attractive candidates for new diagnostic biomarkers of CAG. However, the abnormal expression pattern of lncRNAs in CAG has not been fully elucidated.

In this study, the expression profiles of lncRNAs and mRNAs in CAG were explored by high-throughput RNA sequencing. We identified differentially expressed lncRNAs and mRNAs between CAG and CNAG samples. Based on this information, the lncRNA–mRNA co-expression network in CAG was constructed. This study might provide helpful information for the identification of potential biomarkers for the diagnosis of CAG.

## Materials and Methods

### Collection of Clinical Specimens

A total of 40 patients, including 20 CNAG and 20 CAG patients, were enrolled in this study. All patients were recruited from the China-Japan Union Hospital of Jilin University. All subjects provided their informed consent for inclusion before they participated in this study. This study was conducted in accordance with the Declaration of Helsinki, and the protocol was approved by the Ethics Committee of Jilin University (approval number: 2019082110). The biopsies of the clinical specimens of the outpatients and inpatients were collected when they received the endoscopic procedure in our hospital. The specimens were promptly frozen in liquid nitrogen and then stored at −80°C. The diagnosis based on the collected tissue samples was confirmed by histology.

### Isolation of Total RNA

Total RNA was isolated from tissue samples of 20 CAG and 20 CNAG patients using TRIzol reagent (Invitrogen, Carlsbad, CA, United States), according to the manufacturer’s protocol. The integrity of RNA isolated from the tissue samples was evaluated by standard denaturing agarose gel electrophoresis. The concentration of each RNA sample was determined by analysis with a NanoDropND-1000 spectrophotometer (Agilent, Wilmington, DE, United States). The RNA of each sample was divided into two parts; one part was used for transcriptome sequencing, while the other part was used for quantitative real-time polymerase chain reaction (qRT-PCR).

### High-Throughput Whole-Transcriptome Sequencing

RNA library preparation and RNA library sequencing were performed by Cloud-Seq Biotech (Shanghai, China). In this study, a special design was made by mixing four consecutive specimens of 20 CAG patients to form one group sample (five groups in total), due to the small size of the available specimens. For the same reason, other five group samples were prepared from 20 CNAG patients. Prior to the construction of the RNA libraries, the Ribo-Zero rRNA Removal Kits (Illumina, San Diego, CA, United States) was used to remove the rRNA. Then, rRNA-depleted RNA was used to construct the RNA libraries with a TruSeq Stranded RNA Library Prep Kit (Illumina, San Diego, CA, United States). Briefly, the cDNA fragments were then end-repaired, dA-tailed, and adapter ligated. The ligated cDNA products were purified and treated with uracil DNA glycosylase to remove the second-strand cDNA while keeping first-strand cDNA that was subjected to 14 cycles of PCR amplification. The library quality was evaluated with the Bioanalyzer 2100 system (Agilent Technologies, Richardson, TX, United States). Then, 10 pM libraries were denatured into single-stranded DNA molecules, captured on Illumina flow cells, amplified *in situ* as clusters, and finally sequenced for 150 cycles on Illumina HiSeq Sequencer according to the manufacturer’s instructions. The sequenced reads were disconnected by cutadapt (Kechin et al., 2017) software (v1.9.3), and the low-quality reads were removed to obtain high-quality reads. The high-quality reads were matched to the human genome version HG19 from the UCSC database, and each measured lncRNA was annotated according to the corresponding genome annotation file.

### Identification of Differentially Expressed lncRNAs

High throughput sequencing of RNA-Seq samples was performed by Cloud-Seq Biotech (Shanghai, China). The sequencing procedure was completed on an Illumine Hiseq instrument with 150 bp paired end reads. To perform the transcriptome profiling, the raw sequencing data (high-quality reads) were analyzed by comparing the similarity (alignment) of a reference genome, i.e., the human reference genome (UCSC hg19) using hisat2 software. Transcripts of the lncRNAs and mRNAs were assembled, of which relative abundance of genes was estimated by Cuffdiff software (v2.2.1, part of cufflinks^1^) in the fragments per kilobase of transcript per million mapped reads. Before performing differential expression analysis, we conducted principal component analysis (PCA). The PCA plot of gene expression reveals the data reproducibility in five CAG and five CNAG group (Supplementary Figure 1). The significance of the changes in the expression profile of the transcripts between groups was determined by a statistical test, by which the differentially expressed genes, either lncRNAs or mRNAs, were identified. Furthermore, the target genes of lncRNA were also predicted by their locations to nearby genes. When the two groups of samples are compared, the volcano map is drawn by Python through multiple changes and *p*-value. The threshold set for the significantly differentially expressed lncRNAs was a fold change of ≥2.0 and a *p*-value of < 0.05.

### Functional Enrichment Analysis

Geneontology^2^ is an online analytical tool used to extract comprehensive biological information associated with large candidate gene lists. Gene ontology (GO) analysis of the target genes of the differentially expressed lncRNAs was performed in this study. By bioinformatics analysis, GO terms were selected from the significantly enriched gene sets (*p* < 0.05). The top 10 enriched GO terms between the CAG and CNAG groups with *p* < 0.05 were considered significantly enriched in CAG.

### Construction of the lncRNA–mRNA Network

A co-expression network of lncRNA–mRNA transcripts was constructed to investigate the latent functions of the differentially expressed lncRNAs and the interactions between mRNAs and lncRNAs. The Pearson correlation coefficient (PCC) was calculated to assess the correlation between the differentially expressed lncRNAs and mRNAs. A statistically significant correlation pair was indicated by PCC > 0.99 or *p* < −0.99 and *p* < 0.01. The co-expression network showing the significant pairs was constructed using Cytoscape (version 3.7.2; Smoot et al., 2011) software.

### Protein–Protein Interaction Network Analysis

Metascape^3^ is an online analytical tool used for gene annotation and analysis. The protein–protein interaction (PPI) network analysis was performed in this study using Metascape. By uploading the upregulated mRNA lists and selecting the correct species (*Homo sapiens*), analysis of the PPI network was performed, followed by MCODE (version 1.5.1) algorithm analysis to identify the connected network components. Then, a biological process enrichment analysis was applied to each MCODE component independently. We retained the four best-scoring (by *p*-value) terms as the functional description of the resulting modules.

### qRT-PCR

The SYBR green qRT-PCR assay was used to confirm the differentially expressed lncRNAs identified by high-throughput whole-transcriptome sequencing. Tissue samples were collected from 20 CAG and 20 CNAG patients. The total RNA isolated from the tissue sample of an individual patient was used for reverse transcription and PCR. qRT-PCR was performed using the SYBR green assay kit (TaKaRa Biotechnology, Dalian, China) and a Roche LightCycler 480 instrument (Roche Applied Science, Mannheim, Germany). The five specific primers used for qRT-PCR are listed in Supplementary Table 1. The qRT-PCR program was as follows: denaturation at 95°C for 30 s, followed by 40 cycles at 95°C for 5s, and 60°C for 30 s. Each sample was run in triplicate. The relative amount of lncRNA was normalized against that of GAPDH, and the fold change for each lncRNA was calculated by the 2^–ΔΔ*Ct*^ method.

### Statistical Analysis

Data were expressed as the mean ± standard deviation. The fold change value and *p*-value were used to evaluate the differentially expressed lncRNAs and mRNAs. The significance of the difference of the expression levels of lncRNAs and mRNAs between the CAG and CNAG groups was estimated using the Student’s *t*-test. The false discovery rate was calculated to correct the *p*-value. *p* < 0.05 was regarded as statistically significant.

## Results

### Clinical Features and Diagnosis of Patients With CAG or CNAG

**FIGURE 1:**
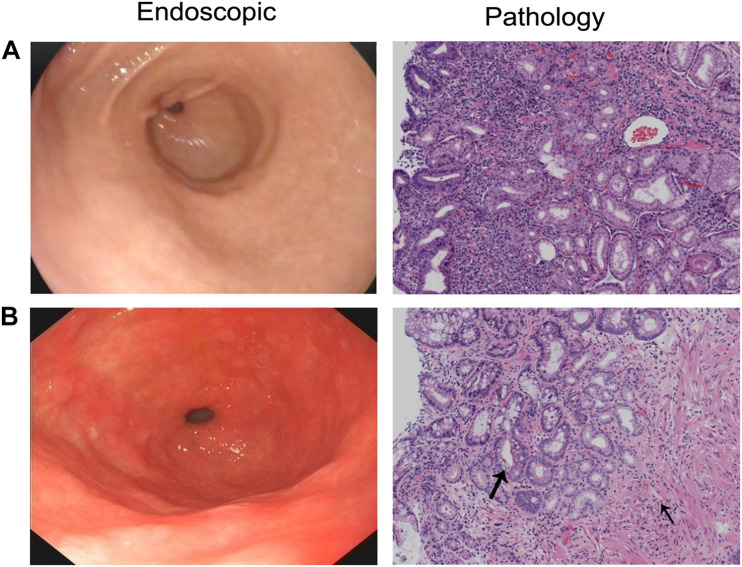
Endoscopic and pathological diagnosis of CAG or CNAG pathological findings of **(A)** CNAG and **(B)** CAG. Loss of gastric glandular cells in the gastric mucosa is indicated by a thin arrow, and replacement with intestinal-type epithelium is indicated by a thick arrow (hematoxylin–eosin staining; 100 × magnification).

This study included 20 patients with CAG (12 males and eight females, mean age of 59 years) and 20 patients with CNAG (11 males and nine females, mean age of 43 years). Between the CAG and CNAG groups, the ratios of cigarette smoking and alcohol drinking were not statistically different. The *Helicobacter pylori* infection rates were 75 and 35% in the CAG and CNAG groups, respectively (Table 1). As shown in Figure 1, the diagnosis of CNAG or CAG was confirmed based on the latest endoscopic examination and pathological diagnosis at the time of recruitment into this study.

**TABLE 1:** Clinical features of the CAG and CNAG patients.

	CAG patients *n* = 20	CNAG patients *n* = 20	*p*
Age, year, median, range	59 (52–69)	43 (35–62)	<0.05
Gender			
Male	12	11	>0.05
Female	8	9	>0.05
Smoking habit	9 (45%)	5 (25%)	>0.05
Drinking habit	12 (60%)	8 (40%)	>0.05
*Helicobacter pylori*	15 (75%)	7 (35%)	<0.05

### Comparison of lncRNAs and mRNAs

**FIGURE 2:**
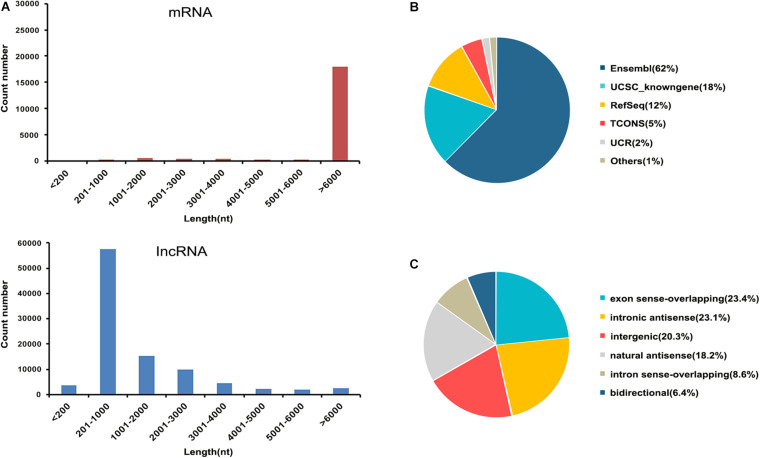
Characteristics of lncRNAs and mRNAs identified in this study. **(A)** Distribution of the transcript lengths of the lncRNAs and mRNAs. **(B)** Pie chart showing the comparative numbers of lncRNAs from authoritative databases. **(C)** Pie chart showing the components of lncRNAs in each category according to their relative chromosomal position to coding genes.

In this study, transcripts obtained from the sequencing data without coding potential were considered as the candidate set of lncRNAs. A total of 97,286 lncRNAs were obtained from all samples (Supplementary Table 2). We systematically analyzed the expression characteristics of these lncRNA and mRNA regarding their distribution in terms of length. All lncRNAs ranged in length from 80 to more than 6,000 nt, and most of the lncRNA were 200–3,000 nt long. All mRNA ranged in length from 80 to more than 6,000 nt, and most of the mRNAs were more than 6,000 nt (Figure 2A). These were collected from the authoritative database Ensembl, UCSC knownGene, RefSeq, TCONS, and UCR as well as some data reported in the literature (Figure 2B). According to the relative chromosomal position of the coding gene, lncRNAs can be classified into six broad categories: exonsense-overlapping, intronic antisense, intergenic, natural antisense, intron sense-overlapping, and bidirectional (Guo et al., 2018). The 97,282 lncRNAs included 22,777 exon sense-overlapping (23%), 22,440 intronic antisense (23%), 19,759 intergenic lncRNAs (20%), 17,672 natural antisense (18%), 8,357 intron sense-overlapping (9%), and 6,280 bidirectional lncRNAs (7%; Figure 2C).

### Identification of Differentially Expressed lncRNAs in CAG

**FIGURE 3:**
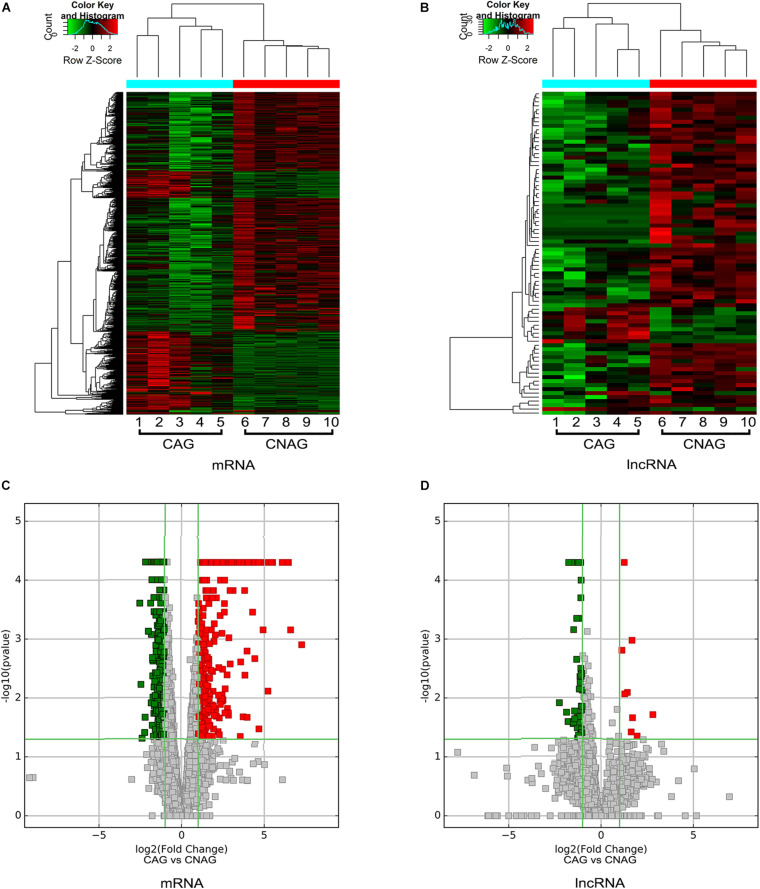
Hierarchical clustering and volcano plots. **(A,B)** Hierarchical clustering shows distinguishable expression profiles of lncRNAs and mRNAs in the CAG and CNAG tissue samples. Upregulated expression is indicated in red, and downregulated expression is indicated in green. **(C,D)** Volcano plots reveal the differentially expressed mRNAs and lncRNAs in the CAG tissue samples compared to the CNAG tissue samples. Upregulated expression is indicated in red, and downregulated expression is indicated in green. *x* axis in the graph represents the fold change (log2 scaled). The *y* axis represents the *p*-value. The red dot represents the differentially expressed genes that are significantly upregulated, and the green dot represents the significantly downregulated differentially expressed genes. The green line is a dividing line between differential and non-differential genes.

We also identified differentially expressed lncRNAs and mRNAs between CAG and CNAG samples, which are shown by hierarchical clustering in Figures 3A,B. In total, 377 upregulated and 763 downregulated mRNAs in CAG were identified by the comparison of CAG and CNAG, using the following thresholds: fold change ≥ 2.0 and *p* < 0.05 (Figure 3C). In addition, 50 upregulated and 66 downregulated lncRNAs in CAG were identified (*p* < 0.05, fold change ≥ 2; Figure 3D). Furthermore, the top 20 differentially expressed lncRNAs in CAG are listed in Table 2.

**TABLE 2:** The top 20 differently expressed lncRNAs in CAG.

Transcript ID	*p*-value	Fold change	LncRNA source	LncRNA_type
ENST00000418770	0.0444	11.9	Ensembl	Bidirectional
TCONS_l2_00013788	0.0192	5.7	TUCP	Intergenic
ENST00000438198	0.0378	5.4	Ensembl	Natural antisense
ENST00000500112	0.0011	5.0	Ensembl	Intergenic
ENST00000559946	0.0086	4.3	Ensembl	Intergenic
ENST00000608442	0.0217	4.3	Ensembl	Natural antisense
ENST00000422847	0.0157	4.2	Ensembl	Intergenic
ENST00000427153	0.0081	3.6	Ensembl	Intergenic
NR_117090	0.0001	3.3	RefSeq	Exonsense-overlapping
uc031rip.1	0.0445	3.2	UCSC_knowngene	Exonsense-overlapping
uc010cdn.3	0.0443	−21.4	UCSC_knowngene	Exonsense-overlapping
ENST00000452120	0.0259	−5.8	Ensembl	Natural antisense
ENST00000565024	0.0072	−5.1	Ensembl	Intergenic
uc002bhh.5	0.0386	−4.4	UCSC_knowngene	Intron sense-overlapping
ENST00000537498	0.01235	−4.3	Ensembl	Intergenic
uc003tnq.3	0.01795	−4.2	UCSC_knowngene	Exonsense-overlapping
NR_027383_2	0.0022	−3.9	RefSeq	Exonsense-overlapping
ENST00000423278	0.02335	−3.8	Ensembl	Intergenic
ENST00000304751	0.02795	−3.7	Ensembl	Intergenic
ENST00000512035	0.02115	−3.7	Ensembl	Intergenic

### Functional Analysis of mRNAs and lncRNAs

**FIGURE 4:**
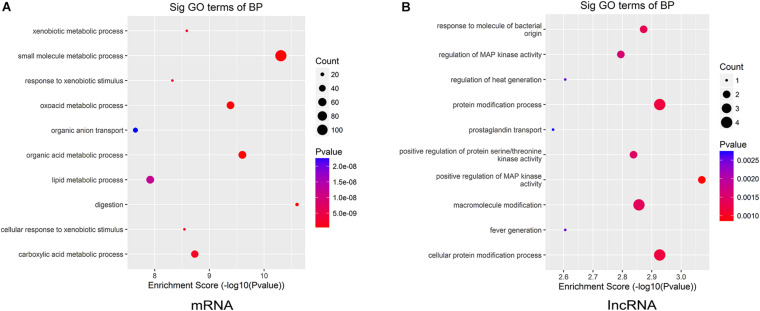
Gene ontology (GO) enrichment analysis. The biological processes of the upregulated mRNAs and lncRNAs were analyzed by GO annotations. **(A)** The top 10 GO terms of the differentially expressed mRNAs in CAG. **(B)** The top 10 GO terms of the differentially expressed lncRNAs in CAG.

To further illustrate the functions of the differentially expressed lncRNAs and mRNAs in CAG, we analyzed the GO categories using a tool called Geneontology, by which the most significant GO terms of upregulated mRNAs and lncRNAs can be found. GO analysis showed that the differentially expressed mRNAs in CAG were significantly enriched in the following four biological processes: small-molecule metabolic processes, xenobiotic metabolic processes, response to xenobiotic stimuli, and oxoacid metabolic processes (Figure 4A). Meanwhile, the top four biological processes related to the differentially expressed lncRNAs in CAG were response to a molecule of bacterial origin, regulation of MAP kinase activity, regulation of heat generation, and protein modification processes (Figure 4B).

### Analysis of Co-Expressed lncRNAs and mRNAs in CAG

**FIGURE 5:**
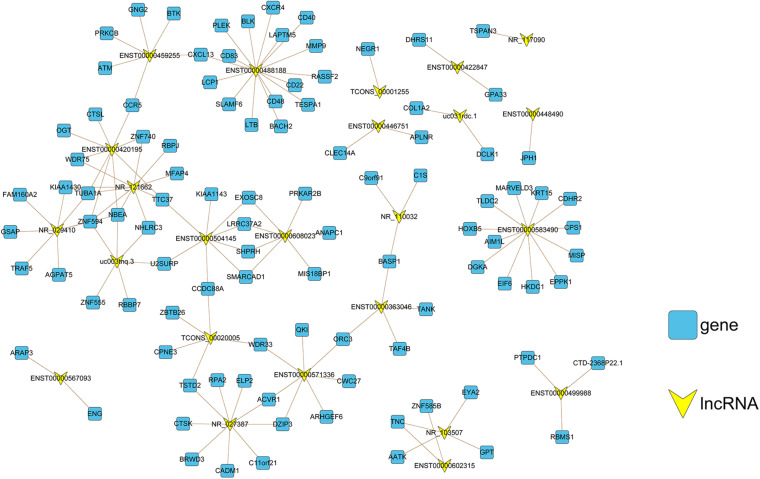
Co-expression network construction. The lncRNA–mRNA co-expression network comprised 130 connections between 24 lncRNAs (yellow nodes) and 101 mRNAs (blue nodes). For the correlation between lncRNAs and mRNAs, the absolute value of the Pearson correlation coefficient was ≥0.98 and *p* < 0.001.

The functions of lncRNAs were analyzed based on the lncRNA–mRNA co-expression network. Here, we used PCC, a co-expression measure for constructing a co-expression network for both upregulated mRNAs and lncRNAs in CAG (Figure 5). Our analysis revealed that ENST00000488188, ENST00000583490, and NR_121662 were the three lncRNAs with the most frequent interactions; therefore, they should be considered as the most active lncRNAs in CAG.

### Gene Regulation by Differentially Expressed lncRNAs

**FIGURE 6:**
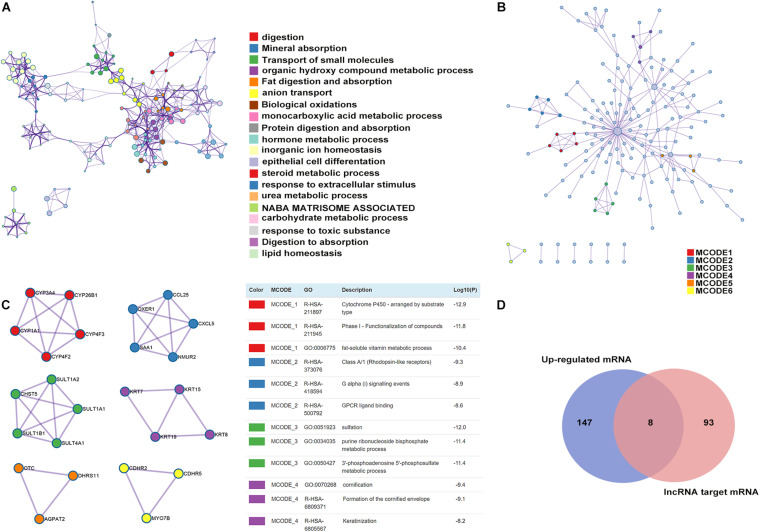
Enrichment analysis, protein–protein interaction (PPI) network construction, and module analysis. **(A)** Metascape visualization of the networks of the top 20 clusters: each node represents one enriched term colored by the cluster ID, and the nodes that share the same cluster are typically close to each other. The size of the node is proportional to the number of input genes falling into that term. Thicker edges represent a higher similarity. **(B)** PPI network construction of upregulated mRNAs: the nodes represent proteins encoded by genes, and the edges represent connections between the nodes. **(C)** Four subnetworks were identified by Cytoscape MCODE plug-in analysis. See Supplementary Table 3 for more details. **(D)** Venn diagram of the differentially expressed lncRNA-targeting genes and mRNAs in CAG.

To predict the protein function using the platform of the PPI network, we uploaded the list of 377 upregulated mRNAs into Metascape (Tripathi et al., 2015) software for functional enrichment analysis. The analytical results showed that the upregulated mRNAs were mainly enriched in biological processes, such as digestion, epithelial cell differentiation, and response to an extracellular stimulus. Most enriched clusters were associated with CAG. The top 20 clusters of significantly enriched terms are listed in Figure 6A. Furthermore, we used the Metascape database, a powerful PPI network, to investigate the interactions between the proteins encoded by the 155 upregulated mRNAs in CAG (Figure 6B). We identified four significant modules via cluster analysis of the PPI network using an analysis tool, called Cytoscape Molecular Complex Detection (MCODE) plugin (Bandettini et al., 2012), which is based on the degree of importance (Figure 6C and Supplementary Table 3). These four identified modules were selected for further GO enrichment analyses of the mRNAs (Figure 6C). We found that the mRNAs in Module 1 were mainly enriched in cytochrome P450, arranged by substrate type, phase I functionalization of compounds, and the fat-soluble vitamin metabolic process. The genes in Module 2 were mainly correlated with class A/1 (Rhodopsin-like receptors), G alpha (i) signaling events, and GPCR ligand binding. The genes in Module 3 were mainly enriched in sulfation, purine ribonucleoside bisphosphate, and the 3′-phosphoadenosine 5′-phosphosulfate metabolic process. The genes in Module 4 were mainly correlated with cornification, formation of the cornified envelope, and keratinization (Figure 6C). Among these differentially expressed mRNAs, those co-expressed with differentially expressed lncRNAs in CAG were chosen as our targets for analysis of PPIs. As shown in Figure 6D, by Venn diagram analysis using the Metascape database, eight mRNAs were found at the intersection of the 155 mRNAs, including DGKA, EIF6, HKDC1, DHRS11, CPS1, KRT15, TESPA1, and CDHR2 as well as 101 lncRNA-targeting genes.

### Expression Validation of lncRNAs

**FIGURE 7:**
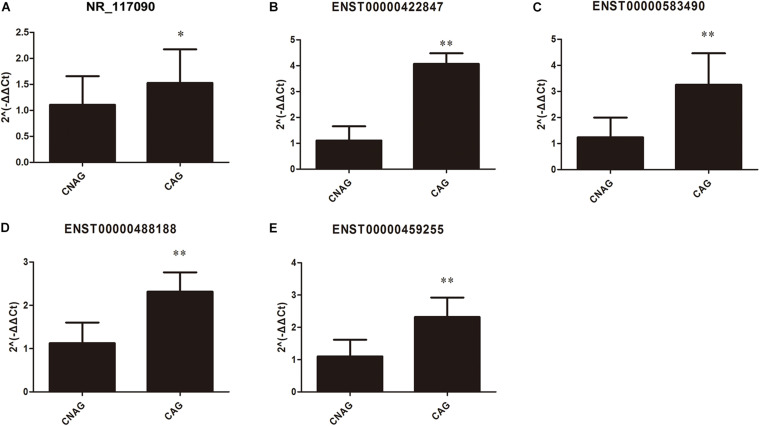
Validation of the differentially expressed lncRNAs in CAG by qRT-PCR. The expression levels of lncRNAs in CAG and CNAG tissue samples, including **(A)** ENST00000422847; **(B)** NR_117090; **(C)** ENST00000583490; **(D)** ENST00000488188; and **(E)** ENST00000459255. All data (mean ± SD) were obtained from 20 experiments (*n* = 20). Statistical analysis was performed using the unpaired *t*-test. ^∗^Represents *p* < 0.05, and ^∗∗^Represents *p* < 0.01.

As mentioned above, these mRNAs were not only upregulated in CAG but also might potentially regulate gene expression. qRT-PCR was used to validate the expression levels of the differentially expressed lncRNAs that were identified by high-throughput sequencing and bioinformatics analysis of 20 CAG and 20 CNAG tissues samples. Five differentially expressed lncRNAs (Figure 7) were selected for verification. As shown in Figure 7, NR_117090, ENST00000422847, ENST00000583490, ENST00000488188, and ENST00000459255 were upregulated in CAG, compared with CNAG. The verification results by qRT-PCR were consistent with data from high-throughput sequencing and bioinformatics analysis.

## Discussion

Worldwide, GC is the fifth most common cancer and the third leading cause of cancer deaths, and the incidence of GC is more than one million cases every year (Bray et al., 2018). Notably, GC is the second leading cause of cancer deaths in China (Chen et al., 2016). Recent studies have revealed that CAG may be a precancerous condition of GC. In addition, it has been reported that the risk of GC development increases significantly with the severity of the precancerous condition at the primary diagnosis (de Vries et al., 2008). Therefore, the early detection of such precancerous lesions is crucial for the early diagnosis of GC (Dinis-Ribeiro et al., 2012). Moreover, the diagnosis of CAG currently relies on histopathological guidance after random endoscopic biopsy sampling (e.g., Sydney biopsy strategy). However, this approach has obvious drawbacks, such as inadequate diagnosis, poor repeatability, and poor correlation between endoscopy and histology. Thus, it is necessary to discover novel sensitive and reliable biomarkers for the development of new methods for the early diagnosis of CAG.

In recent years, lncRNAs have received increasing attention because they have been shown to be involved in tumorigenesis and tumor progression. Much evidence supports that dysregulated lncRNA expression is related to the phenotype and growth characteristics of tumors and is associated with the clinicopathological features and prognosis of cancer patients. It also has been reported that lncRNAs play an important role in the pathogenesis of GC (Lin et al., 2014). According to studies on lncRNAs in GC and CAG, there is one report showing that single-nucleotide polymorphisms (SNPs) interact with PGC, a coding gene with its adjacent lncRNAs, which could affect a person’s susceptibility to GC and CAG (Lv et al., 2018). In that study, 15 pairwise interacting PGC-lncRNA SNPs were found, in which five pairs were relevant to CAG risk. However, to date, the expression profiles of lncRNAs in CAG remain unclear.

To this end, our study investigated the expression profiles of lncRNAs and mRNAs in CAG by high-throughput whole-transcriptome sequencing using tissue samples collected from CAG and CNAG patients. A total of 40 patients (20 CAG and 20 CNAG) were recruited in this study. All of the patients had an endoscopy and a histopathological diagnosis. To explore the potential biological functions of the lncRNAs, the lncRNA–mRNA co-expression network was analyzed through the connection of PPIs.

To the best of our knowledge, this is the first report of high-throughput sequencing to analyze the expression profiles of lncRNAs and mRNAs in CAG and CNAG. By analyzing the sequencing data, we obtained 97,282 lncRNA transcripts and 20,307 mRNA transcripts. The results of transcriptome sequencing revealed that compared with the expression profiles of both the lncRNAs and mRNAs in the CAG and CNAG tissue samples, there were 377 upregulated and 763 downregulated mRNAs as well as 55 upregulated and 66 downregulated lncRNAs in CAG. Most of the differentially expressed lncRNAs in CAG have not been given official names, and their functions remain unknown.

Further analysis revealed that 101 differentially expressed mRNAs were co-expressed with 24 differentially expressed lncRNAs. To predict the latent functions of these lncRNAs, analysis with Geneontology was performed. Among these GO terms, the MAP kinase signaling pathway is reported to play a critical role in the development of CAG and is associated with GC caused by CAG (Li et al., 2018). The MAP kinase signaling pathway is one of the most important GO terms identified by GO analysis, suggesting that regulation of MAP kinase activity by the differentially expressed lncRNAs and mRNAs might contribute to the development of CAG. Another top GO term is lipid metabolic processes. It has been reported that lipid disturbances play key roles in CAG development (Liu et al., 2019).

To better understand the potential interactions and related functions of the co-expressed lncRNAs and mRNAs validated in this study, we combined the co-expressed genes and PPIs to construct an integrated network. A total of eight mRNAs common in both the co-expression network and the PPI network were selected via Venn analysis, including DGKA, EIF6, HKDC1, DHRS11, CPS1, KRT15, TESPA1, and CDHR2. These mRNAs have been reported to be associated with the development of CAG and GC. Among these mRNAs, DGKA has been speculated to serve as a potential biomarker for the diagnosis of GC (Kong et al., 2016). KRT15 was identified in this study and has been related to the prognosis of GC patients by others (Zhang et al., 2019) because of its role in the DNA methylation mechanism of GC. Therefore, KRT15 is a promising prognostic marker for GC. This study had some limitations that must be addressed. For example, a small number of tissue samples were analyzed (only 20 CAG and 20 CNAG samples). The valuable genes and pathways could be further identified from our database. Therefore, we need to enlarge the sample size for further studies. Another defect of this study is that *H*. *pylori* infection is not included in the influencing factors; therefore, this factor will be included in the experimental design in the follow-up study. The present study is our preliminary work. Although plentiful results were obtained from the bioinformatics analysis and transcriptome sequencing in this study, the identification of functional pathways and mechanisms of action related to the differentially expressed genes by experimental approaches is needed in future studies.

## Conclusion

In conclusion, the present study revealed five differentially expressed lncRNAs in CAG that were found by high-throughput whole-transcriptome sequencing and confirmed by qRT-PCR, including NR_117090, ENST00000422847, ENST00000583490, ENST00000488188, and ENST00000459255. These lncRNAs may participate in the development of CAG and may be used as potential biomarkers for the diagnosis of CAG. In addition, our results may be helpful to better understand the potential functions of lncRNAs in CAG. However, further studies are still needed to evaluate the clinical value of these lncRNAs.

## Data Availability Statement

The datasets generated for this study can be found in the NCBI GEO accession GSE153224.

## Ethics Statement

The studies involving human participants were reviewed and approved by Ethics Committee of Jilin University. The patients/participants provided their written informed consent to participate in this study.

## Author Contributions

BZ and LY conceived and supervised the study. BZ and JJ designed experiments. LW and YC performed experiments. XJ and JJ analyzed data. YC wrote the manuscript. BZ made manuscript revisions. All authors read and approved the final manuscript.

## Conflict of Interest

The authors declare that the research was conducted in the absence of any commercial or financial relationships that could be construed as a potential conflict of interest.

## Footnotes

1 http://cufflinks.cbcb.umd.edu/

2 http://www.geneontology.org

3 http://metascape.org

## Supplementary Material

Supplementary Figure 1 Principal component analysis (PCA). The PCA plot of gene expression revealing the data reproducibility in five CAG and five CNAG group.

Supplementary Table 1 Primers used for qRT-PCR.
