# Supplementary figures and images for: Transcriptome Analysis of lncRNA–mRNA Interactions in Chronic Atrophic Gastritis
Source: Front Genet. 2021 Jan 11;11:612951. doi: 10.3389/fgene.2020.612951 (PMC7831747; doi:10.3389/fgene.2020.612951)

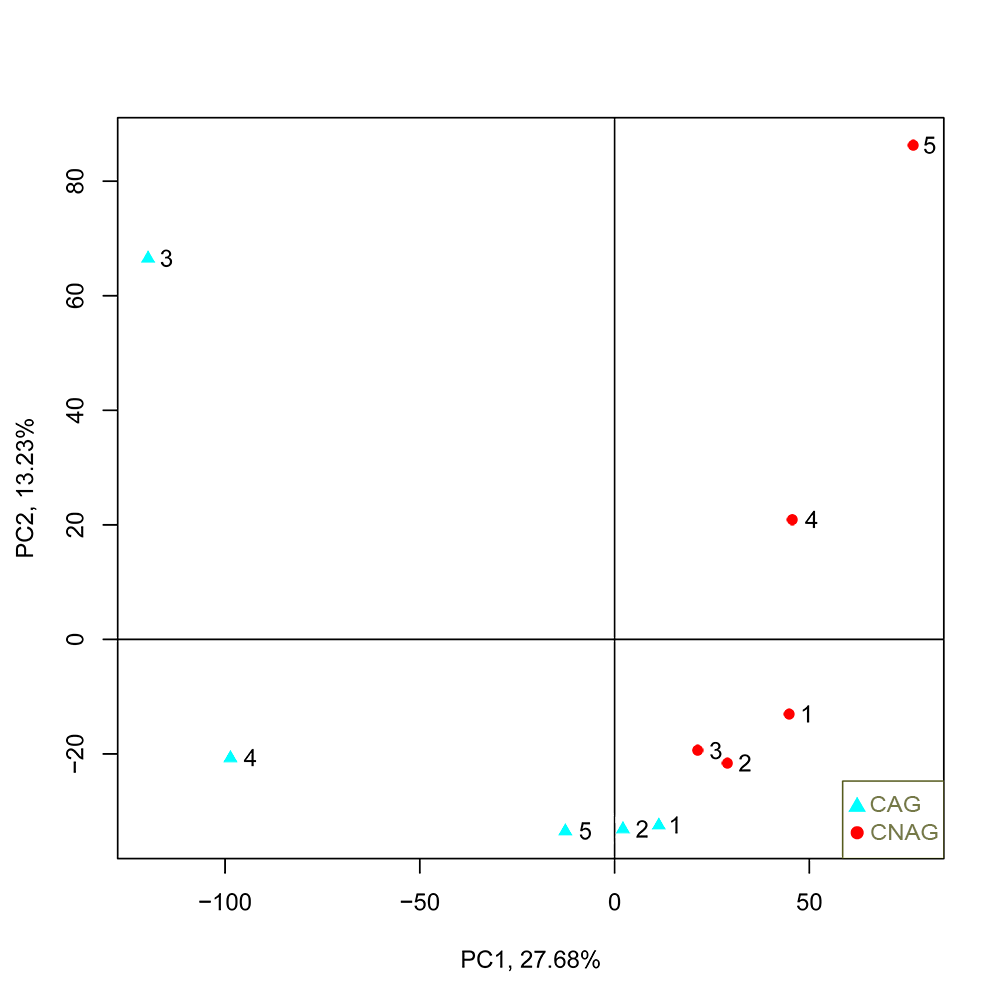

Supplement: Supplementary Figure 1 — Principal component analysis (PCA). The PCA plot of gene expression revealing the data reproducibility in five CAG and five CNAG group. [file Image_1.TIF]
