# Supplementary material for: Transcriptome Analysis of lncRNA–mRNA Interactions in Chronic Atrophic Gastritis
Source: Front Genet. 2021 Jan 11;11:612951. doi: 10.3389/fgene.2020.612951 (PMC7831747; doi:10.3389/fgene.2020.612951)
Supplement: Supplementary Table 1 — Primers used for qRT-PCR. [file Table_1.DOCX]

Supplementary Material

# Supplementary Data Table

**Supplementary Table S1.** Primers used for qRT-PCR

| **Ensembl_Gene_ID** | **Sequence (5’→3’)** | **Product (bp)** |
| --- | --- | --- |
| ENST00000583490 | F: GAAGAGATAGCCAGGCGTCC | 148 |
|  | R: ACCCATTTCCGTCAGTGCTT |  |
| ENST00000422847.1 | F: TGAGACATGTTGCAGACCCC | 113 |
|  | R: TCTTCTTGCGCTTCGTCAGT |  |
| ENST00000459255.1 | F: CTTGGTGGGCGATACAGAGT | 187 |
|  | R: CTTTAGGACCCTTGGCCCTG |  |
| NR_117090 | F: AGTGTACCATTTGCCTCCCG | 156 |
|  | R: TCAGGAGTTCGAAACCAGCC |  |
| ENST00000488188.2 | F: ACCCAGACTGTGGTTTTGCA | 114 |
|  | R: TCATCCACAGACCACACGTG |  |
